# NutriCatt Protocol Improves Body Composition and Clinical Outcomes in Elderly Patients Undergoing Colorectal Surgery in ERAS Program: A Retrospective Cohort Study

**DOI:** 10.3390/nu13061781

**Published:** 2021-05-23

**Authors:** Emanuele Rinninella, Alberto Biondi, Marco Cintoni, Pauline Raoul, Francesca Scialanga, Eleonora Persichetti, Gabriele Pulcini, Roberto Pezzuto, Roberto Persiani, Domenico D’Ugo, Antonio Gasbarrini, Maria Cristina Mele

**Affiliations:** 1 UOC di Nutrizione Clinica, Dipartimento di Scienze Mediche e Chirurgiche, Fondazione Policlinico Universitario A. Gemelli IRCCS, Largo A. Gemelli 8, 00168 Rome, Italy; francesca.scialanga@policlinicogemelli.it; 2 UOC di Chirurgia Generale, Dipartimento di Scienze Mediche e Chirurgiche, Fondazione Policlinico Universitario A. Gemelli IRCCS, Largo A. Gemelli 8, 00168 Rome, Italy; alberto.biondi@policlinicogemelli.it (A.B.); robertopezzuto@gmail.com (R.P.); roberto.persiani@unicatt.it (R.P.); domenico.dugo@unicatt.it (D.D.); 3 Scuola di Specializzazione in Scienza dell’Alimentazione, Università di Roma Tor Vergata, Via Montpellier 1, 00133 Rome, Italy; marco.cintoni@gmail.com; 4 Dipartimento di Medicina e Chirurgia Traslazionale, Università Cattolica Del Sacro Cuore, Largo F. Vito 1, 00168 Rome, Italy; pauline.raoul1@gmail.com (P.R.); antonio.gasbarrini@unicatt.it (A.G.); mariacristina.mele@unicatt.it (M.C.M.); 5 UOSD di Nutrizione Avanzata in Oncologia, Dipartimento di Scienze Mediche e Chirurgiche, Fondazione Policlinico Universitario A. Gemelli IRCCS, Largo A. Gemelli 8, 00168 Rome, Italy; persichettiele@gmail.com (E.P.); gabriele.pulcini@guest.policlinicogemelli.it (G.P.); 6 UOC di Medicina Interna e Gastroenterologia, Dipartimento di Scienze Mediche e Chirurgiche, Fondazione Policlinico Universitario A. Gemelli IRCCS, Largo A. Gemelli 8, 00168 Rome, Italy

**Keywords:** nutritional support, colorectal surgery, elderly, body composition, BIA, phase angle, postoperative complications, length of hospital stay, ERAS program, personalized medicine

## Abstract

Background: A poor body composition, often found in elderly patients, negatively impacts perioperative outcomes. We evaluated the effect of a perioperative nutritional protocol (NutriCatt) on body composition and clinical outcomes in a cohort of elderly patients undergoing colorectal surgery in a high-volume center adopting the ERAS program. Methods: 302 out of 332 elderly (>75 years) patients from 2015 to 2020 were identified. Patients were divided according to their adherence, into “NutriCatt + ERAS” (*n* = 166) or “standard ERAS” patients (*n* = 136). Anthropometric and bioelectrical impedance analysis data were evaluated for NutriCatt + ERAS patients. Complications, length of hospital stay (LOS), and other postoperative outcomes were compared between both groups. Results: In NutriCatt + ERAS patients, significant improvements of phase angle (pre-admission vs. admission 4.61 ± 0.79 vs. 4.84 ± 0.85; *p* = 0.001; pre-admission vs. discharge 4.61 ± 0.79 vs. 5.85 ± 0.73; *p* = 0.0002) and body cell mass (pre-admission vs. admission 22.4 ± 5.6 vs. 23.2 ± 5.7; *p* = 0.03; pre-admission vs. discharge 22.4 ± 5.6 vs. 23.1 ± 5.8; *p* = 0.02) were shown. NutriCatt + ERAS patients reported reduced LOS (*p* = 0.03) and severe complications (*p* = 0.03) compared to standard ERAS patients. A regression analysis confirmed the protective effect of the NutriCatt protocol on severe complications (OR 0.10, 95% CI 0.01–0.56; *p* = 0.009). Conclusions: The NutriCatt protocol improves clinical outcomes in elderly patients and should be recommended in ERAS colorectal surgery.

## 1. Introduction

In recent years, the number of elderly patients admitted to hospital for surgery has increased. This is particularly reported in colorectal surgery; indeed, the prevalence of colorectal cancer (CRC) is rising among older adults [1]. Surgical outcomes could be hampered by a poor physical status. Indeed, aging itself is characterized by progressive physiological and behavioral changes, worsening body composition and body cell mass. [2]. Moreover, the burden of chronic diseases and repeated hospital admission may further worsen nutritional status. The incidence of malnutrition ranges from 12% to 50% among the hospitalized elderly population [3]. In these settings, cancer and oncologic treatments may have an additional negative effect, increasing energy expenditure and leading to weight and skeletal muscle loss, due to a dysregulation of the immune response and metabolic derangements [4]. All these aspects are at the basis of the so-called “disease-related malnutrition” (DRM) [5]. As regards CRC, it is known to affect the intake and absorption of nutrients, leading to increased nutrient requirements, nutrient loss, poor nutrient absorption, or a combination of these factors [6]. Studies have shown that malnutrition is generally associated with a longer length of hospital stay (LOS), and increased morbidity and mortality in cancer patients [7]. Hence, when elderly patients are hospitalized for surgery, they become at risk of a multitude of poor outcomes, including increased mortality, prolonged LOS, high rates of re-admission, skilled nursing facility placement, and functional decline [8]. 

The Enhanced Recovery After Surgery (ERAS) program, first introduced by Kehlet et al. for colorectal surgery [9], aims to reduce the metabolic response to surgery and to improve outcomes in terms of recovery and discharge, postoperative morbidity, and hospital re-admissions [10,11]. To date, ERAS recommendations are standardized according to international guidelines in different surgical areas. Until now, few postoperative dietetic items were introduced into ERAS protocol, leading to better management of postoperative hyperglycemia, insulin resistance, and catabolic response to stress [12]. However, the role of nutrition in reducing a surgically induced stress response, embedded into a multimodal program called the “prehabilitation phase” has also been shown [13]. To make it effective in the hospital practice, in January 2016, our group launched the perioperative NutriCatt protocol within the ERAS program. NutriCatt protocol consists of a perioperative nutritional support program from pre-admission to discharge [14]. The NutriCatt protocol aims to detect the risk of malnutrition early, through Nutritional Risk Screening (NRS-2002), to assess nutritional status through the use of anthropometric and bioelectrical impedance analysis (BIA) data, and to provide adequate personalized nutritional support before and during the hospital stay, in line with international guidelines on nutrition in cancer patients [15]. Compared with the standard ERAS program, the NutriCatt + ERAS protocol achieved better clinical and cost-effectiveness outcomes in a cohort of adult patients undergoing colorectal surgery [14]. However, to date, little is known about the impact of a nutritional support among elderly patients undergoing colorectal surgery.

Aims of the present study were:

- to evaluate the effects of the NutriCatt protocol on the body composition of elderly (>75 years) patients undergoing colorectal surgery from the pre-admission phase, to admission and discharge.
- to compare clinical outcomes such as postoperative complications, re-operation, 30-day mortality, admission to Intensive Care Unit (ICU), and LOS between elderly patients receiving or not the perioperative nutritional support (both within the ERAS program).

## 2. Materials and Methods

### 2.1. Study Design and Ethical Committee Approval

This was a single-center retrospective cohort study of elderly adults (>75 years old) undergoing colorectal surgery for cancer or benign diseases. Patients had been admitted for colorectal surgery from January 2015 to July 2020 at the Fondazione Policlinico Agostino Gemelli IRCCS, Rome, Italy. The study was conducted based on the Helsinki Declaration and according to good clinical practice, following the ERAS program on colorectal surgery [16] and ESPEN guidelines on nutrition in surgery [17]. The study was approved by the Ethical Committee of Fondazione Policlinico A. Gemelli IRCCS-Catholic University of the Sacred Heart (Prot. 50958/17 (4876/18) ID: 1808). All participants signed a consent form recording their agreement to take part in the study and to have the results published. This study was reported according to the STROBE guidelines for cohort studies [18].

### 2.2. Patients

All the following inclusion criteria were used to identify patients eligible for this study: (1) patients > 75 years of age; (2) patients undergoing colorectal surgery; (3) patients treated according to the ERAS program; (4) patients having a compliance >70% to the ERAS program. Adherence to the ERAS program was assessed with a physician-reporting questionnaire and calculated as the number of pre- and intra-operative interventions fulfilled out of 14 (the number of protocol items included), similar to other studies reported in the literature [19].

Patients’ baseline characteristics were collected by the hospital staff: sex, age, height, body weight, body mass index (BMI), NRS-2002 score, diagnosis, American Society of Anesthesiology (ASA) score, Charlson Comorbidity Index (CCI), neoadjuvant chemotherapy (NAD), surgery type, TNM stage, and time of surgical intervention, as well as wrist, arm, waist and hips circumferences. During the hospital stay, the surgical, anesthesiologic, and nursing staff remained unchanged.

### 2.3. NutriCatt Nutritional Protocol 

The perioperative nutritional protocol, called NutriCatt, included in the ERAS program for colorectal surgery, is detailed in our previous study [14]. It consists of five phases: (i) “pre-admission phase” (approximately 3 weeks before admission) including the scoring of NRS-2002, anthropometric measurements, nutritional counselling and the delivery of a personalized diet; (ii) “admission phase” including a nutritional re-evaluation and the assessment of adherence with the pre-admission diet through a patient-reported questionnaire; (iii) “postoperative phase” providing three progressive diets from the first postoperative day; (iv) “discharge phase” including anthropometric and BIA measurements, and the prescription of a personalized diet with an ONS supplementation (if necessary) for the first 2 weeks after discharge; and finally (v) a one-month outpatient visit and nutritional counselling. 

### 2.4. BIA Measurement Protocol

BIA was performed with BIA 101 (Akern, Florence, Italy) at 50 kHz frequency. Patients were told to abstain from eating a meal and drinking large amounts of fluid at least 2 to 3 h before the test. They were asked to remove any jewelry. Patients were made to lie down supine on a bed. The subjects were asked to separate the legs from 30° to 40°. Electrodes were applied under aseptic conditions on the right side, with injecting electrodes respectively placed on the dorsum of hand and feet on the metacarpal and metatarsals, and reading electrodes placed between the medial and lateral malleolus of the same side. The reading electrodes of the wrist were placed between the radial styloid and ulnar prominence of the wrist. The distance between injecting and reading electrodes was 5 cm. Single measurements were reported by dietitians’ staff. Body composition data, such as fat-free mass (FFM), body cellular mass (BCM), body cellular mass index (BCMI), and total body water (TBW) were obtained using BODYGRAM™ software (Akern, Florence, Italy).

### 2.5. Discharge Criteria

All the patients were discharged if they met all the following predefined discharge criteria: tolerance to solid food, autonomous mobilization for >6 h or return to baseline conditions before surgery, adequate pain control (VAS < 4) with oral medications, bowel recovery (time to first flatus or stool) and no evidence of postoperative complications.

### 2.6. Outcomes

The following outcomes were assessed:

- Within patients undergoing NutriCatt protocol: body composition changes between pre-admission, admission, and discharge including FFM (kg), phase angle (PhA; degree), resistance (Rz; Ohm), reactance (Xc; Ohm), BCM (kg), BCMI, and TBW (L);
- Between patients undergoing NutriCatt protocol + ERAS and those in standard ERAS:
  ○ LOS defined as the number of days from surgery to discharge calculated from discharge letters;
  ○ Number and type of postoperative complications according to Clavien–Dindo staging [20];
  ○ Number of severe complications (Clavien–Dindo grade ≥ III);
  ○ 30-day mortality defined as death occurring within 30 days of colorectal surgery;
  ○ Number of re-operations;
  ○ Number of admissions to Intensive Care Unit (ICU).

### 2.7. Data Collection and Statistical Analysis

Baseline characteristics of the patients, type of intervention, and surgical outcomes were extracted from an Excel^©^ (Microsoft Office, Washington, DC, USA) database provided by the General Surgery Unit and analyzed retrospectively. The dietary and medical staff of the Clinical Nutrition Unit prospectively collected the data concerning the nutritional status of the patients adhering to the NutriCatt protocol. 

Statistical analysis was conducted using STATA^®^ software (Version 14.0, Stata Corporation; College Station, TX, USA). The normal distribution of the variables was tested with the Shapiro–Wilk test. The Chi-square test was used to compare categorical variables. Continuous variables are expressed as mean and SD, and dichotomous ones as absolute frequency and percentage. To detect statistically significant differences between various groups for the continuous variables, the Student’s *t*-test was used, while for the dichotomous variables, the Chi-square test or Fisher’s Exact test was used where necessary. A *p*-value < 0.05 was considered statistically significant. A univariate analysis was carried out for postoperative outcomes. Moreover, the odds ratios (ORs) and 95% confidence intervals (95% CIs) were estimated to evaluate the association between the nutritional intervention (NutriCatt protocol) and postoperative outcomes. To rule out other confounding factors, multiple logistic regression analysis was used. Given the retrospective nature of the study, a post-hoc analysis of sample power was carried out to test the quality of the data obtained.

## 3. Results

### 3.1. Patients Baseline Characteristics

**Figure 1:**
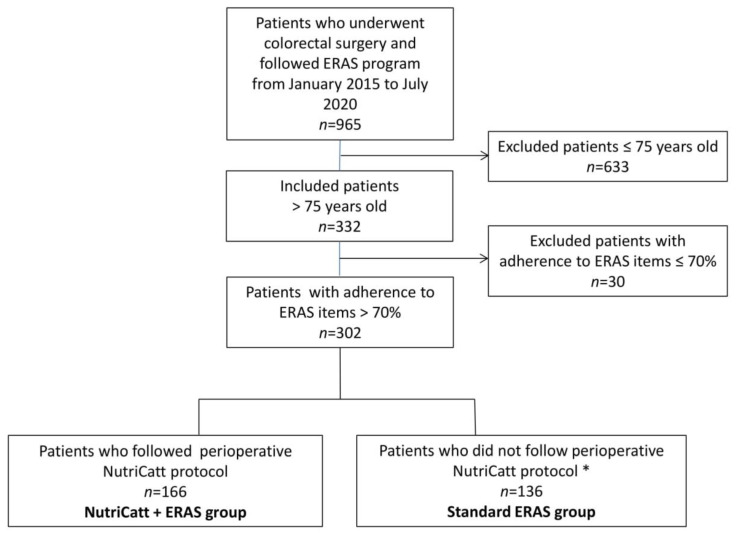
Flowchart of the retrospective cohort study. Abbreviations: ERAS, Enhanced Recovery after Surgery. * for the following reasons: failure to undergo a pre-hospitalization nutritional check, fast-track regimen, emergency intervention.

Between January 2015 and July 2020, 965 patients underwent colorectal surgery. Of a total of 332 patients >75 years of age, 302 patients met the inclusion criteria and were retrospectively included. Of these, 166 (55%) underwent NutriCatt protocol within the ERAS program, while 136 patients (45%) followed the standard ERAS program for several reasons: failure to undergo a pre-hospitalization nutritional check, fast-track regimen, emergency intervention. Figure 1 illustrated the flowchart of the study. 

Of the total of patients, 154 (51%) were men. The mean age was 80.47 ± 4.08 years. The main diagnosis was cancer (90.4% of cases). There were no significant differences in terms of baseline characteristics (age, gender, diagnosis, CCI, surgery type, TNM stage, NAD, time of surgical intervention) between the two groups, except for ASA score ≥ 3 (*p* = 0.002) and type of surgery (*p* = 0.04). Table 1 details the characteristics of patients.

Table 2 details the baseline nutritional characteristics of the NutriCatt + ERAS group. Of the 166 patients enrolled in the nutritional protocol, the mean BMI was 26.5 ± 3.9 kg/m^2^. Patients at risk of malnutrition (according to NRS-2002) were 44.5%.

### 3.2. Body Composition Changes between Pre-Admission, Admission, and Discharge in Patients following NutriCatt Protocol

All the patients undergoing nutritional protocol reported at least a moderate (>50%) or satisfactory (>75%) adherence to dietary prescriptions. A complete nutritional evaluation—at pre-admission (T0), admission (T1), and discharge (T2)—was performed in 96 patients (Table 3). There were no significant changes in terms of body weight, BMI, Rz, TBW, FFM across the periods. However, PhA significantly increased at admission compared to pre-admission (4.84 ± 0.85 vs. 4.61 ± 0.79; *p* = 0.001), and still more at discharge compared to pre-admission (5.85 ± 0.73 vs. 4.61 ± 0.79; *p* = 0.0002). Likewise, BCM was significantly higher at admission and discharge than at pre-admission. The mean time between T0 and T1 was 32.1 ± 37.4 days.

### 3.3. Impact of NutriCatt Protocol on Outcomes of Hospitalization

**Figure 2:**
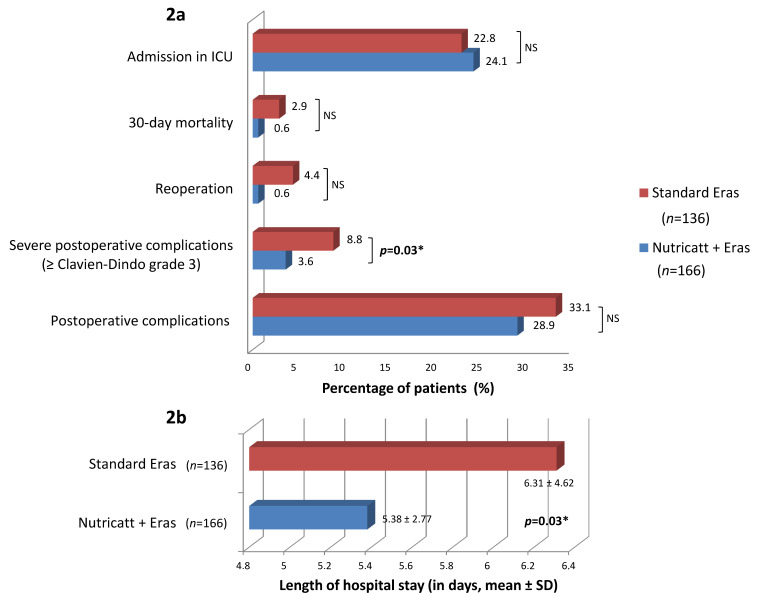
Associations between protocol types and postoperative outcomes (**2a**) and length of hospital stay (**2b**). Abbreviations: Eras, Enhanced Recovery after Surgery; ICU, intensive care unit; NS, non-significant (*p* ≥ 0.05); SD, standard deviation. * statistically significant (*p* < 0.05).

The NutriCatt + ERAS group experienced significantly less severe complications (≥grade 3) than the standard ERAS group (6 vs. 12; *p* = 0.03). The severe complications (Clavien–Dindo grade ≥ III) were evisceration (*n* = 1), perforations (*n* = 3), coronary heart disease (*n* = 1), pneumonia (*n* = 1), pleural effusion (*n* = 1), intra-abdominal abscesses (*n* = 2), anastomotic leak (*n* = 8), myocardial infarction (*n* = 1), atrial fibrillation (*n* = 1), intestinal obstruction (*n* = 1), multi-organ failure (*n* = 1), and dehydration (*n* = 1). Moreover, LOS was significantly shorter in the NutriCatt + ERAS group compared with standard ERAS (5.38 ± 2.77 vs. 6.31 ± 4.62; *p* = 0.03). Conversely, the number of complications, re-operation, admission to ICU, and 30-day death did not significantly differ between the NutriCatt + ERAS and standard ERAS groups (Figure 2). 

A post-hoc power analysis was carried out on the incidence of severe complications in the two groups. The incidences of 12.5% and 26.7% were found in the two groups, respectively, with a number of 166 and 136 patients. α type error I was 0.05 and the power of the study was 87.8% (β: 0.122).

In the multiple logistic regression analysis, patients following the NutriCatt + ERAS protocol showed a lower risk for severe complications than the standard ERAS group (OR = 0.10; 95% CI 0.01–0.56; *p* = 0.009; Table 4). Conversely, no significant differences were shown between the NutriCatt + ERAS and the standard ERAS groups in terms of LOS (OR = 0.53; 95% CI 0.19–1.43; *p* > 0.05; Table 4). No associations were found between age, gender, CCI, surgery type, tumor stage with severe complications, and longer LOS (>5 days) except for ASA score with LOS > 5 days.

## 4. Discussion

This retrospective cohort study shows that perioperative nutritional support is effective in improving body composition (PhA and BCM) from pre-admission to discharge in elderly patients (over 75 years old) undergoing colorectal surgery, according to ERAS protocol. Moreover, patients pre-treated with nutritional support experienced a lower rate of severe postoperative complications and a lower LOS compared with those treated with standard ERAS protocol. A multiple regression analysis confirmed a significant protective effect of nutritional support on severe complications. 

To date, little is known about the impact of personalized perioperative nutritional support on body composition in elderly patients undergoing surgery. It is well-known that the elderly patient is at greater risk of frailty due to age-related consequences and comorbidities. Patients over 75 years of age often experience reductions of up to 60% in muscle strength and 30% in physical function compared with younger patients [21]. Indeed, aging has been associated with a reduced muscle protein synthetic response to protein intake, known as anabolic resistance [22]. In this study, the average age of patients undergoing surgery was 80, an age particularly at risk for sarcopenia. An apparent contradiction could be noted between the mean BMI of 26.5 kg/m^2^ (interpretable as overweight in absence of other measures of nutritional status) and the high risk of malnutrition (NRS-2002 ≥ 3), present in almost half of patients. These results highlight the importance of screening nutritional status with validated easy nutritional assessment tools, such as NRS-2002 score, and not only with BMI calculation. 

To assess nutritional status, various body composition measurement methods have been validated including BIA, dual-energy X-ray, magnetic resonance imaging, and body composition computed tomography. Among them, BIA is an easy-to-use, non-invasive, and reproducible technique for evaluating changes in body composition. BIA has been validated for the assessment of body composition and nutritional status in cancer patients [5]. PhA, one of the main BIA parameters, reflects the relative contributions of fluid (resistance) and cellular membranes (capacitance) of the human body, and has been used as a nutritional prognostic factor in several clinical contexts [23]. A lower PhA suggests cell death or decreased cell integrity, whereas a higher PhA suggests large quantities of intact cell membranes [16]. A recent systematic review evaluating 13 studies (7668 subjects) showed a strong correlation between PhA and sarcopenia: PhA is lower in sarcopenic subjects and, in turn, the prevalence of sarcopenia is higher in subjects with a low PhA [23]. An original recent study confirmed that PhA is related to sarcopenia in older men (>65 years of age) with cancer [24]. Given these reasons, PhA appears to be a reliable marker of nutritional status in older patients. Moreover, to date, the BIA method is widespread in clinical practice and is considered a safe and low-cost tool for evaluating body composition.

Most of the patients present in our cohort (86.1%) were affected by neoplastic disease. Malnutrition is a common issue among patients with cancer, affecting up to 85% of patients in certain cancers [25]. In severe cases, malnutrition can lead to cachexia, characterized by a loss of lean body mass, muscle wasting, and impaired immune, physical and mental function [4]. Cancer cachexia is associated with poor response to therapy, increased susceptibility to treatment-related adverse events, and poor survival outcomes [26]. The present study shows that nutritional perioperative intervention can significantly improve—even in the elderly and cancer population—many functional indices of body composition, such as PhA and BCM. Indeed, we can hypothesize that a high dietary protein content of the preoperative diet, according to ESPEN guidelines [15], might alleviate the loss in cellular integrity, and therefore would delay or reduce body composition derangements in colorectal patients [27].

This study also found that personalized nutritional support significantly decreased the number of severe postoperative complications in elderly patients undergoing colorectal surgery. These results are in line with our previous findings in surgical patients [14,28], and could be shareable in several other surgical contexts. Indeed, our team have shown that the NutriCatt protocol in the ERAS program reduces LOS without increasing the risk of postoperative complications in patients undergoing liver resections [28]. Another study [29] evaluated the impact of the ERAS program (including an early postoperative feeding) on clinical outcomes in elderly patients, reporting a reduction in LOS and an improvement in short-term postoperative complications. These results are relevant in this type of population, since advanced age has been associated with increased postoperative morbidity and mortality due to reduced organ function or reserve and a trend of higher ASA scores and CCI [8,30].

In this study, a higher ASA score was shown to be associated with a longer LOS. These findings are consistent with the existing literature. Indeed, the correlation of ASA scores with operating times, LOS, postoperative infection rates, overall morbidity, and mortality rates has been demonstrated in patients with adenocarcinoma of the gastroesophageal junction [31], in gynecological surgery patients [32], and in patients undergoing elective colorectal resection [33]. Surprisingly, in our study, the rate of ASA score ≥3 was significantly greater in the NutriCatt + ERAS cohort. This could probably explain the lack of association between NutriCatt protocol and LOS at the multiple regression analysis. Another hypothesis could be the already short LOS (mean 5.81 days) in the entire population due to the application of ERAS protocol. 

Furthermore, although few studies reported that overall compliance to ERAS protocol decreased with increased patient age [34,35], this study reported high compliance results regarding the adherence to NutriCatt protocol (>50%) for all enrolled patients from pre-admission to discharge. This highlights that NutriCatt protocol is feasible in elderly patients and could be systematically recommended for them in a clinical context, in which malnutrition is often overlooked. 

This study has some limitations. First, this is a retrospective, single-center study with lack of randomization. Appropriate randomization and a prospective model would have made the study more reliable from a statistical point of view, even though this would make it less ethical towards patients. However, we strived to compare two similar populations, since one cohort followed an ERAS + NutriCatt protocol and one cohort an ERAS standard protocol without nutritional prehabilitation. The characteristics of the two cohorts did not vary significantly in terms of age and comorbidities; they were only different in terms of surgical risk (greater in the NutriCatt cohort) and type of surgery (higher rate of laparoscopic surgery in the NutriCatt cohort). However, the type of surgery did not correlate with severe complication and LOS at the multivariable regression analysis. To assess the statistical power of the study in inferential terms, the quality of the data obtained was also tested through a post-hoc analysis of sample power. Furthermore, body weight, BMI and body composition were not measured in the standard ERAS group; indeed, due to the retrospective nature of the study, these data are lacking because nutritional intervention was provided, as previously described. Prospective studies are needed to investigate the differences in body weight, BMI, body composition between perioperative nutritional interventions + ERAS protocol and the standard ERAS protocol. Moreover, the number of patients having complete nutritional data both at pre-hospitalization and discharge was reduced due to the clinical and management framework in which we operate. Finally, the term “elderly” does not have a standardized definition as regards the surgical risk; we chose 75 years of age as an arbitrary cut-off, according to previously published studies [29,36,37]. 

## 5. Conclusions

In conclusion, this study showed that the NutriCatt protocol, with its preoperative personalized nutritional support, could improve PhA and BCM parameters in patients over 75 years. Moreover, when it is applied within the ERAS program, undergoing colorectal surgery, it is an effective and feasible strategy to reduce severe postoperative complications. Further studies are needed to better investigate the impact of this protocol on the nutritional status in other surgical settings, in order to improve the awareness of the value of a personalized nutritional support in perioperative care. 

## Figures and Tables

**Table 1:** Patient characteristics.

	All (*n* = 302)	NutriCatt *+* ERAS (*n* = 166)	Standard ERAS (*n* = 136)	*p*-Value
Age (years), mean ± SD	80.47 ± 4.08	80.76 ± 4.05	80.12 ± 4.06	0.18
Gender (*n*, %)				0.28
Male	154 (51.0)	80	74	
Female	148 (49.0)	86	62	
Diagnosis (*n*, %)				0.12
Adenocarcinoma	273 (90.4)	157	116	
Adenoma	10 (3.3)	5	5	
Diverticulitis	3 (0.9)	0	3	
Other	16 (5.3)	4	12	
Site of Surgery (*n*, %)				0.39
Right hemicolectomy	119 (39.4)	64	55	
Left hemicolectomy	57 (18.9)	32	25	
Rectal surgery	102 (33.8)	58	44	
Other	24 (7.9)	12	12	
TNM stage (*n*, %)				0.32
0	18(5.9)	14	4	
I	53(17.7)	25	28	
IIA	64(21.2)	40	24	
IIB	21(6.9)	10	11	
IIC	2(0.6)	2	0	
IIIA	5(1.7)	3	2	
IIIB	58(19.2)	31	27	
IIIC	10(3.3)	6	4	
IV	13(4.3)	6	7	
NR	58(19.2)	29	29	
NAD (*n*, %)				0.55
Yes	35 (11.6)	18	17	
No	267 (88.4)	148	119	
CCI, mean ± SD	5.50 ± 2.46	5.71 ± 2.24	5.24 ± 2.69	0.10
ASA Score (*n*, %)				0.002 *
≥3	101 (33.5)	64	37	
<3	201 (66.5)	102	99	
Time of surgical intervention (min), mean ± SD	217.2 ± 76.9	211.28 ± 75.52	224.02 ± 78.45	0.15
Type of surgery (*n*, %)				0.04 *
Laparotomy	9 (3.0)	7	2	
Laparoscopy	254 (84.1)	145	109	
Robotic	39 (12.9)	14	25	

Abbreviations: ASA, American Society of Anesthesiology; CCI, Charlson Comorbidity Index; ICU, intensive care unit; NAD, neoadjuvant chemotherapy; NR, not reported; SD, standard deviation. * statistically significant (*p* < 0.05).

**Table 2:** Baseline nutritional characteristics of patients following NutriCatt protocol (*n* = 166).

	Media ± SD	n (%)
Body weight (kg)	66.8 ± 11.2	
Height (cm)	158.9 ± 9.6	
BMI (kg/m^2^)	26.5 ± 3.9	
Wrist circumference (cm)	17.0 ± 1.2	
Arm circumference (cm)	28.1 ± 3.2	
Waist circumference (cm)	93.5 ± 12.9	
Hip circumference (cm)	99.6 ± 8.2	
NRS-2002		
1		5 (3.0)
2		86 (52.5)
3		68 (41.5)
4		5 (3.0)
Rz (Ohm)	498.1 ± 82.8	
Xc (Ohm)	41.5 ± 8.8	
Phase angle (°)	4.7 ± 0.9	
Total body water (L)	37.1 ± 6.9	
Total body water (%)	55.9 ± 7.1	
Body extracellular water (L)	19.4 ± 3.7	
Body extracellular water (%)	52.5 ± 5.3	
Fat-free mass (kg)	49.2 ± 8.7	
Fat-free mass (%)	74.2 ± 8.9	
BCM (kg)	23.1 ± 5.7	
BCMI	9.1 ± 1.8	

Abbreviations: BCM, body cellular mass; BCMI, body cellular mass index; BMI, body mass index; NRS, nutritional risk screening; Rz, resistance; Xc, reactance.

**Table 3:** Body Composition changes between Pre-Admission and Admission in Patients following NutriCatt protocol (*n* = 96).

	Pre-Admission T0	Admission T1	Discharge T2	*p*-Value (ANOVA)	T0–T1	T1–T2	T0–T2
Body weight (kg)	66.4 ± 11.1	66.1 ± 10.9	65.7 ± 10.8	0.39			
BMI (kg/m^2^)	26.5 ± 3.9	26.4 ± 4.1	26.2 ± 4.6	0.42			
Rz (Ohm)	492.6 ± 83.1	495.1 ± 86.6	507.0 ± 90.7	0.56			
Xc (Ohm)	39.4 ± 7.7	42.2 ± 9.7	54.7 ± 8.2	0.04 *			0.04 *
Phase angle (°)	4.61 ± 0.79	4.84 ± 0.85	5.85 ± 0.73	0.002 *	0.001 *		0.0002 *
Total body water (L)	36.9 ± 7.1	36.9 ± 6.8	36.5 ± 7.9	0.84			
Total body water (%)	56.3 ± 7.5	56.6 ± 8.3	56.2 ± 9.0	0.77			
Extracellular water (L)	19.7 ± 3.7	19.2 ± 3.6	19.6 ± 5.4	0.79			
Extracellular water (%)	53.5 ± 4.9	52.2 ± 5.5	52.6 ± 7.4	0.91			
Fat-free mass (kg)	48.9 ± 8.8	49.1 ± 8.5	47.8 ± 11.4	0.12			
Fat-free mass (%)	74.5 ± 9.3	75.3 ± 9.8	72.5 ± 13.0	0.16			
BCM (kg)	22.4 ± 5.6	23.2 ± 5.7	23.1 ± 5.8	0.03 *	0.02 *		
BCMI	8.9 ± 1.8	9.3 ± 1.9	9.2 ± 1.9	0.04 *	0.05 *		

Abbreviations: BCM, body cellular mass; BCMI, body cellular mass index; BMI, body mass index; Rz, resistance; Xc, reactance. * Statistically significant (*p* < 0.05). Data are expressed in mean ± standard deviation.

**Table 4:** Multiple Regression Analysis for Severe Postoperative Complications and LOS > 5 days.

	Severe Complications (*n* = 18)		LOS > 5 Days (*n* = 117)	
	OR (95% CI)	*p*-value	OR (95% CI)	*p*-value
NutriCatt + ERAS vs. standard ERAS	0.10 (0.01–0.56)	0.009 *	0.53 (0.19–1.43)	0.21
Age	1.04 (0.88–1.22)	0.59	0.97 (0.86–1.09)	0.66
Sex	2.67 (0.65–10.89)	0.17	0.97 (0.36–2.55)	0.95
CCI	1.15 (0.86–1.54)	0.35	0.96 (0.76–1.22)	0.76
ASA Score < 3	0.69 (0.13–3.65)	0.64	0.33 (0.11–0.96)	0.04 *
Type of surgery				
Laparotomy	Reference		Reference	
Laparoscopy	0.15 (0.01–2.19)	0.16	0.13 (0.01–1.3)	0.08
Robotic	0.38 (0.02–6.78)	0.51	0.61 (0.05–8.18)	0.71
Tumor stage				
I	Reference		Reference	
II	0.63 (0.11–3.6)	0.61	1.8 (0.53–6.10)	0.34
III	0.72 (0.12–4.29)	0.71	0.63 (0.18–2.15)	0.46
IV	0.70 (0.06–8.37)	0.78	0.69 (0.10–4.61)	0.71
Severe Complications	-	-	3.08 (0.56–17.26)	0.20

Abbreviations: ASA, American Society of Anesthesiology; CCI, Charlson Comorbidity Index; ERAS, Enhanced Recovery After Surgery; LOS, length of hospital stay; OR, odds ratio; vs., versus. * Statistically significant (*p* < 0.05).

## Data Availability

The data presented in this study are available on request from the corresponding author for any academic use upon citation of this article. The data are not publicly available due to privacy and permission restricted to publication of this article only.
